# Axial Thorax-Pelvis Coordination During Gait is not Predictive of Apparent Trunk Stiffness

**DOI:** 10.1038/s41598-018-37549-9

**Published:** 2019-01-31

**Authors:** Maarten R. Prins, Sjoerd M. Bruijn, Onno G. Meijer, Peter van der Wurff, Jaap H. van Dieën

**Affiliations:** 1 Research and Development, Military Rehabilitation Centre ‘Aardenburg’, Doorn, The Netherlands; 2 Department of Human Movement Sciences, Faculty of Behavioural and Movement Sciences, Vrije Universiteit Amsterdam and Amsterdam Movement Sciences, Amsterdam, The Netherlands; 3 Institute for Human Movement Studies, HU University of Applied Sciences Utrecht, Utrecht, The Netherlands; 4 Orthopaedic Biomechanics Laboratory, Fujian Medical University, Quanzhou, Fujian, P.R. China

## Abstract

The coordination of axial thorax and pelvis rotations during gait has been shown to be affected by several pathologies. This has been interpreted as an indication of increased apparent axial trunk stiffness, but arm swing may also affect these rotations. The objectives of this study were to assess the effect of trunk stiffness and arm swing on the relative timing (‘coordination’) between thorax and pelvis rotations, and to assess if apparent trunk stiffness can be inferred from thorax-pelvis kinematics. A forward dynamic model was constructed to estimate apparent trunk stiffness from observed thorax and pelvis rotations and arm swing moment around the longitudinal axis of the trunk of 30 subjects. The effect of independent manipulations of trunk stiffness and arm swing moment on thorax-pelvis coordination and gain of axial thorax-pelvis rotations were assessed using the same forward dynamic model. A linear regression model was constructed to evaluate whether forward dynamic model-based estimates of axial trunk stiffness could be inferred directly from thorax-pelvis rotations. The forward dynamic model revealed that axial trunk stiffness and arm swing moment have opposite effects on axial thorax-pelvis coordination. Apparent axial trunk stiffness could not be predicted from observed thorax-pelvis rotations.

## Introduction

At low gait speed, thorax and pelvis demonstrate synchronous axial rotations (around the vertical axis) in the same direction, i.e., in-phase rotation. When speeding up, this pattern gradually changes towards rotations in opposite directions, i.e., out-of-phase rotation^1^. The *relative phase* between these segment rotations can vary from plus to minus 180 degrees. A value of plus or minus 180 degrees corresponds to perfect out-of-phase rotation and a value of 0 degrees corresponds to perfect in-phase rotation^2^. If timing of thorax rotations is expressed relative to the pelvis (i.e., thorax-pelvis relative phase), negative values indicate that thorax rotations lag pelvis rotations, and positive values indicate that thorax rotations lead those of the pelvis. In healthy individuals, thorax-pelvis relative phase is around minus 20 degrees in slow walking (1 km/h). The lag of thorax phase increases with gait speed resulting in a thorax-pelvis relative phase of minus 150 degrees in fast walking (5.4 km/h)^1^. This relative timing of thorax and pelvis rotations, henceforward ‘axial thorax-pelvis coordination’ can be affected by several pathologies; for example, the shift from in-phase towards out-of-phase coordination was found to be smaller in patients with Parkinson’s disease^2^, stroke^3^, pregnancy related pelvic-girdle pain^4^ and low-back pain^1^ with a 20 to 30 degree more in phase thorax-pelvis coordination at higher gait speeds. This has been interpreted as indicative of an increased axial trunk stiffness in such pathologies^5,6^.

Axial trunk stiffness, in this context, is a parameterization of the relationship between axial rotations of thorax and pelvis and axial trunk moments. Trunk muscles show components of phasic and tonic activity during the gait cycle^7^ that, combined with forces from passive structures, generate the net trunk moment. During gait, this moment shows a close to linear relationship with the angle between thorax and pelvis^6^, as in a torsion spring that pulls thorax and pelvis towards a neutral relative orientation. Consequently, the mechanical behaviour of the trunk around the vertical axis during gait can be parameterized as an *apparent* axial stiffness^6,8,9^.

In addition to apparent axial trunk stiffness^6,9,10^, arm swing amplitude may affect thorax-pelvis kinematics^11–14^, via shoulder reaction forces acting on the thorax. These forces result in a moment around the longitudinal axis of the trunk^6^, henceforward ‘arm swing moment’. An increased arm swing moment would pull the timing of thorax rotations towards arm swing timing, that is, out of phase with axial pelvis rotations. At given arm swing frequency, accelerations of the arms must increase with increasing arm swing amplitude and hence the arm swing moment will increase as well. The axial accelerations of the thorax are driven by the arm swing moment and by the internal trunk moment, which act in opposite directions, limiting axial thorax rotations^6^. With increasing arm swing amplitudes during gait, internal axial trunk moment also increase^15^.

Some insight into the effect of axial trunk stiffness and arm swing on thorax-pelvis rotations in gait of healthy subjects can be obtained from previous studies. Two studies that estimated apparent axial trunk stiffness in healthy subjects’ gait reported that stiffness changes as a function of gait speed^6,9^. However, the fact that trunk stiffness is modulated with gait speed, provides no conclusive evidence that this modulation causes concomitant modifications in thorax-pelvis coordination, because changes in, e.g., step length and/or frequency may influence intersegmental coordination as well^16^. In one study, trunk stiffness of healthy subjects was increased with an orthopaedic brace on the trunk. This resulted in more in-phase thorax-pelvis rotations during gait compared to a condition without the brace^10^. However, this was mainly the result of changes in pelvis timing, whereas changes in thorax-pelvis relative phase in patients are mainly the result of changes in thorax timing^16^. The effect of arm swing was evaluated in a study in which one arm of healthy subjects was constrained. This clearly resulted in more in-phase axial thorax-pelvis coordination at higher gait speeds compared to the normal arm swing condition. However, the timing and amplitude of the kinematics of the unconstrained arm were affected as well^12^, which may have influenced axial thorax-pelvis coordination.

Indications of increased axial trunk stiffness and decreased arm swing amplitude have been observed in some of the pathologies in which axial thorax-pelvis coordination is known to be affected during gait. The relative phase of thorax and pelvis was found to be less variable in gait of patients with Parkinson’s disease^2^ and patients with low-back pain demonstrated lower stride-to-stride variability of axial rotations between thorax and pelvis^17^. Although these less variable coordination patterns could be the result of an increased apparent axial trunk stiffness, they might also be the result of modulation of timing and amplitude of muscle activation to reduce stride-to-stride variability of thorax-pelvis kinematics. External perturbations did not reveal increased apparent axial trunk stiffness in low-back pain patients, who demonstrated more in-phase thorax-pelvis coordination than healthy controls^18^. Arm swing amplitude was found to be lower in gait of patients with Parkinson’s disease^13^ and unilaterally in stroke patients^14^, but arm swing amplitude was found to be unaffected in low-back pain patients with lumbar disc herniation, who did demonstrate more in phase axial thorax-pelvis coordination compared to healthy controls^19^.

Although the aforementioned studies provide some insight into the association between apparent axial trunk stiffness, arm swing and axial thorax-pelvis coordination in healthy subjects and patients, the causality remains elusive. A forward dynamic model could be used to demonstrate how axial trunk stiffness and arm swing moment modulate thorax-pelvis coordination during gait. The first objective of this study, therefore, was to assess the effect of axial trunk stiffness and arm swing moment on axial thorax-pelvis coordination using a forward dynamic model. The second objective was to evaluate the common variance between apparent axial trunk stiffness and arm swing moment. The third objective of this study was to assess whether apparent axial trunk stiffness can be inferred from thorax-pelvis kinematics. We hypothesized that increased axial trunk stiffness and reduced arm swing moment would result in more in-phase rotations of thorax and pelvis. Regarding the second objective, given the associations of both axial trunk stiffness and arm swing moment with gait speed, we hypothesized that a moderate to high positive correlation is present between these two variables, which would confound inferences on axial trunk stiffness from thorax-pelvis kinematics. Given the second hypothesis, estimation of axial trunk stiffness from thorax-pelvis kinematics would be prone to error. Hence, we hypothesized that the predictive value of thorax-pelvis kinematics to estimate axial trunk stiffness would be limited.

## Methods

### Normalization

In this study, experimental data of 30 subjects were used. To reduce between subject variability caused by differences in anatomy some outcomes were normalized^20^. Normalized internal trunk moment, normalized arm swing moment and normalized apparent axial trunk stiffness and damping were corrected for subject height and weight.

### Model description and validation

**Figure 1:**
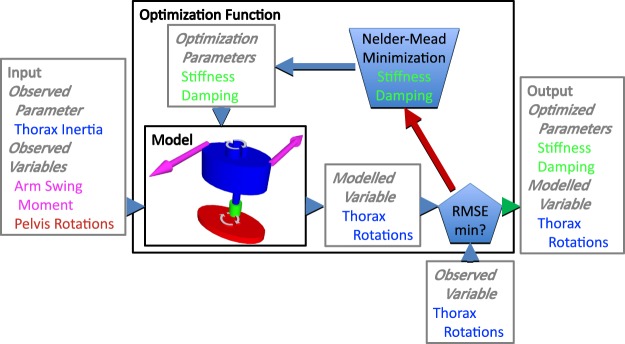
Visual representation of the model and the optimization function. For each subject, the model was run with the (fixed) observed parameter and variables and an initial guess for axial trunk stiffness and damping. Subsequently, axial trunk stiffness and damping were estimated by minimization of the root mean squared error between observed and predicted axial thorax rotations.

We constructed a model of the trunk in MATLAB^®^ Release 2018a using Simscape™ in the Simulink^®^ environment (The Mathworks Inc., Natick, MA, USA). All code and data accompanying this manuscript can be found via the data availability statement. The thorax and pelvis were modelled as two rigid segments with one joint between them, L5S1, which allowed only axial rotation. The model aimed to ‘predict’ a time series of axial thorax rotations based on observed time series of axial pelvis rotations, arm swing moment in terms of time series of the moment caused by shoulder reaction forces around the longitudinal axis of the trunk through L5S1, thorax inertia and estimated axial trunk stiffness and damping. Axial trunk stiffness and damping coefficients were estimated using an optimization procedure that minimized the root mean square error (RMSE) of the observed thorax rotations vs the modelled thorax rotations using a Nelder-Mead minimization procedure, that searches for a local minimum in a two-dimensional space using a triangular simplex^21^. Initial stiffness was set to 100 Nm/rad and initial damping was set to 1 Nms/rad. The termination tolerance for the dependent variable, i.e., the RMSE of thorax rotations, and both independent variables, i.e., stiffness and damping, was set to 10^−4^. Figure 1 gives a visual representation of the model and the optimization function.

Data that were used as input for the model were obtained from a previously published study^18^. In this study 15 healthy subjects and 15 patients with low-back pain were included. Low-back pain patients had to have experienced low-back pain in the three months prior to inclusion and have a minimum current 100 mm Visual Analogue Score (VAS) for low-back pain intensity of 20 mm at the time of inclusion. An overview of subject characteristics is presented in Table 1.

**Table 1:** Subject characteristics.

	Control	CLBP	p
N (m/f)	15 (15/0)	15 (15/0)	—
Age (years)	33 (6)	34 (11)	0.78
Height (cm)	186 (11)	184 (8)	0.55
Weight (kg)	80 (14)	84 (9)	0.33
VAS Pain (mm)	0 (0)	22 (17)	—

The protocol of the previous study was approved by the Medical Ethical Committee of the VU Medical Centre, Amsterdam, The Netherlands (NL 37399.029.12) and was in accordance with the Declaration of Helsinki. An informed consent was obtained from all participants. In the study, fifteen healthy subjects and fifteen subjects with chronic low-back pain walked at a speed of 4 (km/h) on a treadmill for two minutes. This speed is high enough for possible differences in thorax-pelvis coordination between patients and controls to become clearly visible, and not too high for most patients to keep up with the treadmill^1^.

Whole-body kinematics were captured at a rate of 100 (samples/s). Axial pelvis rotations were registered and shoulder reaction forces were calculated using top-down inverse dynamics^22–24^. The inertia of the thorax was estimated from body mass and the circumference and length of the segment^8^. The forward dynamic model was run for each individual, resulting in thirty predicted axial trunk stiffness and damping coefficients and thirty predicted time series of thorax rotations.

For each subject, the goodness of fit of the optimized model prediction was determined in terms of common variance, i.e., *R*^2^, of the observed vs modelled thorax rotations.

To evaluate whether the stiffness values obtained through the optimization procedure were plausible, we estimated stiffness using inverse dynamics as well^6^. Here, stiffness was estimated as the slope of the regression line between the internal axial trunk moment, and axial trunk angle. Trunk moment was calculated using the same principles as used to calculate the shoulder reaction forces^22–24^. We calculated the % common variance and root mean square error between the stiffness estimates obtained with both methods.

The focus of the current study was not to compare the subject groups with and without chronic low-back pain, but to evaluate effects of axial trunk stiffness and arm swing amplitude on axial thorax-pelvis coordination in both groups. Therefore, after checking for group differences in apparent axial trunk stiffness and damping, magnitude of shoulder reaction forces and thorax-pelvis coordination (relative phase and gain, see below) with independent sample t-tests, the data of both groups were pooled.

### How do axial trunk stiffness and arm swing moment affect axial thorax-pelvis coordination?

After estimating the apparent axial trunk stiffness and damping of each subject with the corresponding modelled time series of thorax rotations, the effects of axial trunk stiffness and arm swing moment on axial thorax-pelvis coordination were assessed. To this aim, simulations were run after multiplication of either apparent trunk stiffness or the experimental arm swing moments by factors of 0.5, 0.75, 1.25 and 1.5., assuming that the obtained time series of axial thorax rotations would reflect the effects of decreased or increased axial trunk stiffness or arm swing moment.

The effects of these manipulations on thorax-pelvis coordination were quantified by calculating the phase and gain of the frequency response function from pelvis to thorax rotations at the stride frequency. The stride frequency was determined as the local peak in the power spectrum of the pelvis rotations closest to 1 (Hz). A gain of one reflects equal amplitudes of pelvis and thorax rotations and higher gains correspond to a larger thorax rotation amplitude for a given pelvis rotation amplitude. No statistics were performed on these outcomes, because the multiplication factors were chosen arbitrarily and the level of significance between the different quantities of axial trunk stiffness and arm swing moment depends on the multiplication factors.

### Does arm swing moment confound the association between axial thorax-pelvis coordination and apparent axial trunk stiffness?

The % common variance between the normalized forward dynamic model-based estimates of apparent trunk stiffness and normalized observed arm swing moment was calculated to assess whether arm swing amplitude confounds the association between axial thorax-pelvis coordination and apparent axial trunk stiffness.

### Can observed coordination between axial thorax and pelvis rotations be used to predict apparent axial trunk stiffness?

A linear regression model was constructed to assess whether observed thorax-pelvis coordination can be used to predict normalized forward dynamic model-based estimates of apparent axial trunk stiffness. We tested whether the dependent and independent variables were normally distributed using a Kolmogorov-Smirnov test. Multicollinearity between the independent variables was evaluated by calculating the correlation coefficient. Linearity of the relation between the variables was visually checked using scatter plots. Subsequently, we constructed a linear regression model with thorax-pelvis relative phase and gain and an interaction term as independent variables and apparent axial trunk stiffness as dependent variable. Thorax-pelvis relative phase and gain of each of the thirty subjects were estimated from the observed kinematics. The goodness of fit of the regression models was evaluated by calculating the % common variance between the axial trunk stiffness predicted by the regression model and that returned by the forward dynamic model.

## Results

### Model validation

**Figure 2:**
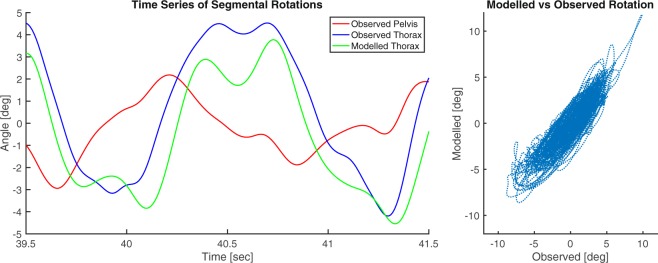
Typical example of observed vs modelled axial thorax rotations. Left: A section of the two-minute time series (39.5–41.5 seconds) of observed axial pelvis rotations and observed and modelled axial thorax rotations of one subject. Right: Observed versus modelled axial thorax rotations over the entire time series of the same subject. In this subject, the common variance was 85%.

A typical example of observed versus modelled axial thorax rotations of one subject is presented in Fig. 2. On average, the % common variance between observed and modelled thorax rotation time series was 83% (SD 13%). We found no significant differences in normalized axial trunk stiffness and damping, normalized arm swing moment or thorax-pelvis relative phase and gain between low-back pain patients and healthy controls. The average stiffness estimate as obtained by the forward dynamic model was 124 (SD 54) (Nm/rad) and that as obtained through inverse dynamics was 101 (SD 37) (Nm/rad). The common variance between these values was 89% (R = 0.94, p < 0.001) and the root mean squared error was 23 (Nm/rad). The estimated stiffness values of all participants using both methods are presented in Supplementary Figure S1.

### How do axial trunk stiffness and arm swing moment affect axial thorax-pelvis coordination?

**Figure 3:**
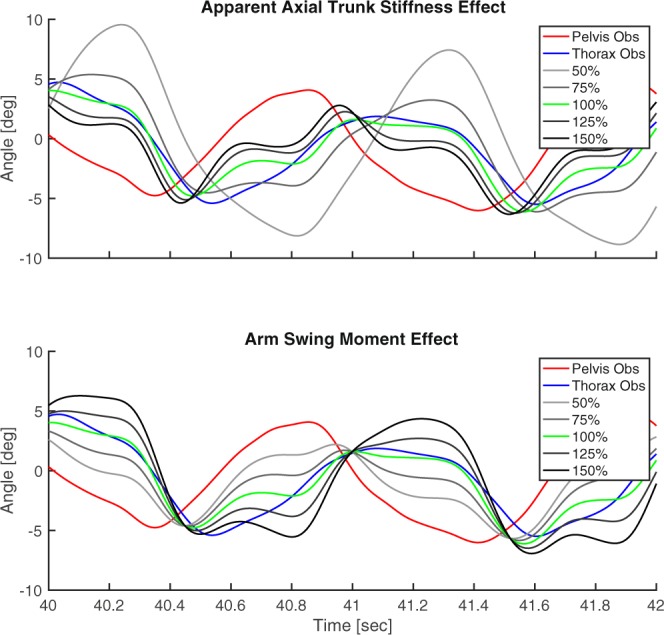
Typical example of the simulated effect of axial trunk stiffness and arm swing moment on axial thorax rotations. Only a section of the two-minute trial is shown (40–42 seconds). A clear effect of both axial trunk stiffness and arm swing moment on thorax-pelvis relative phase and gain is visible. In the upper graph, high axial trunk stiffness (black line) results in more in-phase thorax-pelvis coordination and a smaller amplitude of axial thorax rotations. The opposite effect can be seen for decreased axial trunk stiffness (light grey line). In the lower graph, increased arm swing moment (black line) results in more out-of-phase thorax-pelvis coordination and an increase in thorax rotation amplitude. The opposite effect can be seen for decreased arm swing amplitude (light grey line).

A typical example of the simulated effects of axial trunk stiffness and arm swing moment on thorax-pelvis coordination in one subject is presented in Fig. 3. In this example, a clear effect of both trunk stiffness and arm swing moment on thorax-pelvis relative phase and gain can be observed.

**Figure 4:**
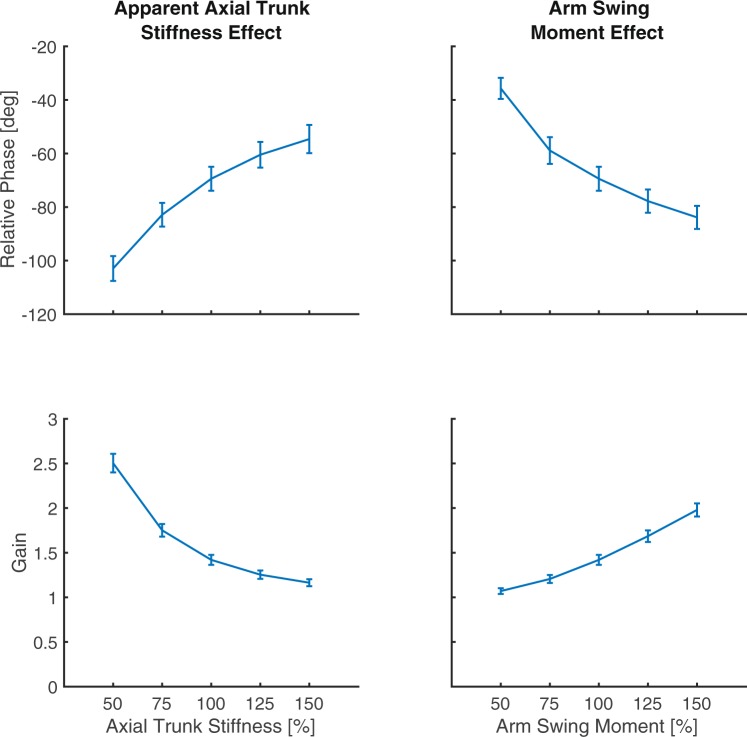
The modelled effects of altered axial trunk stiffness and arm swing moment on thorax-pelvis relative phase and gain. There is a clear positive relationship between axial trunk stiffness and thorax-pelvis relative phase and between arm swing moment and thorax-pelvis gain. A negative relationship is present between axial trunk stiffness and thorax-pelvis gain and between arm swing moment and thorax-pelvis relative phase.

The average modelled effects of axial trunk stiffness and arm swing moment on axial thorax-pelvis coordination are presented in Fig. 4. With increasing axial trunk stiffness, pelvis and thorax become more in-phase and gain decreases. With increasing arm swing moment, pelvis and thorax become more out-of-phase and gain increases.

### Does arm swing moment confound the association between axial thorax-pelvis coordination and apparent axial trunk stiffness?

Observed arm swing moments and forward dynamic model-based estimates of apparent axial trunk stiffness had a common variance of 22% (p = 0.008), indicating that effects of arm swing moment on axial trunk stiffness on axial thorax-pelvis relative phase and gain is small, albeit significant.

### Can observed coordination between axial thorax and pelvis rotations be used to predict apparent axial trunk stiffness?

Normalized stiffness and axial thorax-pelvis relative phase and gain were normally distributed and no collinearity was present. We found no indication for a non-linear relationship between these the dependent and independent variables in the scatterplots. Axial thorax-pelvis relative phase did not contribute significantly to the prediction of apparent axial trunk stiffness (p = 0.99) nor did gain (p = 0.32), or the interaction between phase and gain (p = 0.65).

## Discussion

**Figure 5:**
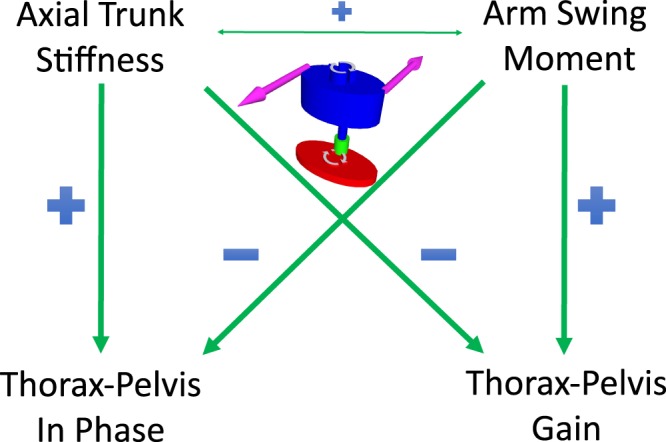
The effect of axial trunk stiffness and arm swing moment on thorax-pelvis relative phase and gain. An increase in axial trunk stiffness results in more in-phase coordination of axial thorax and pelvis rotations and a decrease in thorax-pelvis gain. The opposite effect occurs with increasing arm swing moment. A small but significant correlation is present between axial trunk stiffness and arm swing moment.

The first objective of this study was to assess the effects of axial trunk stiffness and arm swing moment on axial thorax-pelvis coordination using a forward dynamic model. We found, as hypothesized, that both increased axial trunk stiffness and decreased arm swing moment resulted in more in-phase axial rotations of thorax and pelvis as seen in several pathologies^1–3^, and a lower gain (Fig. 5). The second objective was to evaluate the common variance between apparent axial trunk stiffness and arm swing moment. In contrast to our second hypothesis, we found only a low common variance between these outcomes. The third objective of this study was to assess whether apparent axial trunk stiffness can be estimated directly from thorax and pelvis kinematics. In accordance with our expectations, the predictive value of thorax-pelvis kinematics to estimate axial trunk stiffness was limited. However, this appears to be caused only for a small amount by covariance of arm swing moment and axial trunk stiffness.

The forward dynamic trunk model utilized in this study predicted a clear effect of altered axial trunk stiffness on thorax-pelvis coordination. Still, observation of thorax and pelvis kinematics in an individual subjects’ gait did not yield a significant prediction of apparent axial trunk stiffness. We expected observed arm swing moment and estimated apparent axial trunk stiffness to covary which could (partially) explain this lack of predictability of trunk stiffness from thorax-pelvis kinematics. However, we found only a low common variance between these outcomes. Hence, it appears that in healthy subjects and patients with low-back pain, between subject variance in thorax-pelvis coordination is mainly caused by other factors than apparent axial trunk stiffness and arm swing moment.

In previous studies on several pathologies, the relative phase of axial thorax-pelvis rotations during gait was found to be affected^1–4^. However, to the best of our knowledge, the gain between these segments was not reported in these studies. An increased range of motion of axial pelvis rotations has been reported in patients with lumbar disc herniation^19^, pregnancy related pelvic-girdle pain^4^, and in a subgroup of chronic low-back pain patients with relatively in-phase thorax-pelvis coordination^18^. In these studies, thorax range of motion was not found to be significantly different between groups. Higher pelvis range of motion combined with an equal thorax range of motion would imply a lower transfer from axial pelvis rotations to the thorax. Such combined alterations in phase and gain would be in line with increased apparent axial trunk stiffness and/or decreased arm swing amplitude in these patients according to the present study. In the study of Huang *et al*.^19^, no significant differences between patients and controls were reported for arm swing amplitude. This may imply an increased axial stiffness in patients with lumbar disc herniation, but alternative strategies that may alter thorax-pelvis coordination cannot be excluded.

There are some limitations of this study that need to be addressed. First, the axial trunk moment was modelled as if provided by a linear passive spring/damper-system. This was motivated by previous studies in healthy subjects that showed a linear relation between axial trunk angles and moments during gait and, in the present study, resulted in a good fit for thorax rotations in both healthy subjects and patients with low-back pain. However, it is possible that this linear relationship does not exist in patients with altered thorax-pelvis coordination, when they alter timing of trunk muscle activation. In this case their behaviour cannot be modelled as a simple passive system. Studies that estimate trunk moment angle relationships in these patients should be performed to determine if patient data fit the model approach used here, and if such a model is to be used for further comparison of trunk coordination between healthy subjects and patients with altered axial thorax-pelvis coordination. A second limitation is that we evaluated the effect of arm swing moment on thorax-pelvis coordination as a means to assess the effect of increased or decreased arm swing amplitude as observed in some pathologies in which arm swing amplitude is affected^13,14^. Although these variables are related^15^ and we found a high common variance, the relationship is not perfect. Other factors, such as the acceleration and velocity profiles of arm swing could affect arm swing moment as well.

The focus of this study was on the direction of the effect of simulated altered arm swing moment and axial trunk stiffness on predicted thorax-pelvis coordination using a forward dynamic model. The same observed pelvis rotation time series were used within subjects to simulate these effects. We did not assess any possible effect of altered arm swing moment or axial trunk stiffness on pelvis kinematics. The silent assumption that pelvis kinematics would be unaffected is probably unrealistic, but we assume that this had no effect on our hypotheses. Moreover, we did not study the effect of pelvis kinematics on thorax-pelvis coordination. In multiple studies, increased range of motion of axial pelvis rotations was found in combination with more in-phase thorax-pelvis rotation^4,18,19^. Possibly, pelvis range of motion could influence thorax-pelvis relative phase. Alterations in pelvis motion might be caused by altered movement patterns of the lower extremities^25^, and this could cause a change in timing of thorax rotations, but new research is needed to specifically investigate these relationships.

## Conclusion

We conclude that both apparent axial trunk stiffness and arm swing moment clearly affect relative phase and gain of axial thorax-pelvis rotations. The forward dynamic model predicted that increased axial trunk stiffness and decreased arm swing moment result in more in-phase thorax-pelvis rotation, as reported in several patient groups, and a decreased gain. Apparent axial trunk stiffness could not be predicted from axial thorax-pelvis coordination.

## Supplementary Information

Supplementary Figure

## Data Availability

The experimental data is avaiable at 10.17026/dans-xzh-ydsu, the forward dynamic Simulink model, and MATLAB-code used to calculate all presented outcomes and plot Figs 2–4 are available at, https://doi.org/10.17026/dans-28q-v6rg.

## References

[1] Lamoth CJC (2002). Pelvis-thorax coordination in the transverse plane during walking in persons with nonspecific low back pain. Spine (Phila Pa 1976).

[2] Van Emmerik REA, Wagenaar RC, Winogrodzka A, Wolters EC (1999). Identification of axial rigidity during locomotion in parkinson disease. Arch. Phys. Med. Rehabil..

[3] Van Criekinge T (2017). Trunk biomechanics during hemiplegic gait after stroke: A systematic review. Gait Posture.

[4] Wu WH (2008). Gait in pregnancy-related pelvic girdle pain: Amplitudes, timing, and coordination of horizontal trunk rotations. Eur. Spine J..

[5] Lamoth CJ, Meijer OG, Daffertshofer A, Wuisman PI, Beek PJ (2006). Effects of chronic low back pain on trunk coordination and back muscle activity during walking: changes in motor control. Eur Spine J.

[6] Kubo M, Holt KG, Saltzman E, Wagenaar RC (2006). Changes in axial stiffness of the trunk as a function of walking speed. J. Biomech..

[7] Hu H (2012). Control of the lateral abdominal muscles during walking. Hum. Mov. Sci..

[8] Zatsiorsky, V. M. *Kinetics of Human Motion*. (Human Kinetics, 2002).

[9] van Dieën, J. H., Meijer, O. G., Lamoth, C. J. C. & Wu, W. H. Modeling pelvis and thorax rotations in healthy and pathological gait. *Proc*. *ISB XXth Congr*. *29th Annu*. *Meet*. (2005).

[10] Wu WH (2014). Effects of experimentally increased trunk stiffness on thorax and pelvis rotations during walking. Hum. Mov. Sci..

[11] Bruijn SM, Meijer OG, van Dieën JH, Kingma I, Lamoth CJC (2008). Coordination of leg swing, thorax rotations, and pelvis rotations during gait: The organisation of total body angular momentum. Gait Posture.

[12] Ford MP, Wagenaar RC, Newell KM (2007). Arm constraint and walking in healthy adults. Gait Posture.

[13] Roggendorf J (2012). Arm swing asymmetry in Parkinson’s disease measured with ultrasound based motion analysis during treadmill gait. Gait Posture.

[14] Johansson GM, Frykberg GE, Grip H, Broström EW, Häger CK (2014). Assessment of arm movements during gait in stroke - The Arm Posture Score. Gait Posture.

[15] Angelini, L., Damm, P., Zander, T., Arshad, R. & Di, F. Effect of arm swinging on lumbar spine and hip joint forces Effect of arm swinging on lumbar spine and hip joint forces. *J*. *Biomech*, 10.1016/j.jbiomech.2017.09.011 (2017). 10.1016/j.jbiomech.2017.09.011 28941955

[16] Huang Y (2010). The effects of stride length and stride frequency on trunk coordination in human walking. Gait Posture.

[17] van den Hoorn W, Bruijn SM, Meijer OG, Hodges PW, van Dieën JH (2012). Mechanical coupling between transverse plane pelvis and thorax rotations during gait is higher in people with low back pain. J. Biomech..

[18] Prins MR, Van der Wurff P, Meijer OG, Bruijn SM, van Dieën JH (2016). Mechanical perturbations of the walking surface reveal unaltered axial trunk stiffness in chronic low back pain patients. PLoS One.

[19] Huang YP (2011). Gait adaptations in low back pain patients with lumbar disc herniation: Trunk coordination and arm swing. Eur. Spine J..

[20] Hof AL (1996). Scaling gait data to body size. Gait Posture.

[21] Nelder JA, Mead R (1965). A Simplex Method for Function Minimization. Comput. J..

[22] Wu G (2005). ISB recommendation on definitions of joint coordinate systems of various joints for the reporting of human joint motion - Part II: Shoulder, elbow, wrist and hand. J. Biomech..

[23] Wu G (2002). ISB recommendation on definitions of joint coordinate system of various joints for the reporting of human joint motion—part I: ankle, hip, and spine. J. Biomech..

[24] Hof AL (1992). An explicit expression for the moment in multibody systems. J. Biomech..

[25] Müller R, Ertelt T, Blickhan R (2015). Low back pain affects trunk as well as lower limb movements during walking and running. J. Biomech..

