# Supplementary material for: Axial Thorax-Pelvis Coordination During Gait is not Predictive of Apparent Trunk Stiffness
Source: Sci Rep. 2019 Jan 31;9:1066. doi: 10.1038/s41598-018-37549-9 (PMC6355803; doi:10.1038/s41598-018-37549-9)
Supplement: Supplementary file 1 — Supplementary Figure [file 41598_2018_37549_MOESM1_ESM.pdf]

Axial Thorax-Pelvis Coordination During Gait is not Predictive of Apparent Trunk Stiffness

Maarten R. Prins[1,2,3*], Sjoerd M. Bruijn[2,4], Onno G. Meijer[2,4], Peter van der Wurff[1,3], Jaap H. van Dieën[2]

1) Research and Development, Military Rehabilitation Centre 'Aardenburg', Doorn, The Netherlands
2) Department of Human Movement Sciences, Faculty of Behavioural and Movement Sciences, Vrije Universiteit Amsterdam and Amsterdam Movement Sciences, Amsterdam, The Netherlands
3) Institute for Human Movement Studies, HU University of Applied Sciences Utrecht, Utrecht, The Netherlands
4) Orthopaedic Biomechanics Laboratory, Fujian Medical University, Quanzhou, Fujian, PR China

* Corresponding author

E-mail: MR.Prins@MRCDoorn.nl

Supplementary figure 1. Stiffness obtained by forward and inverse dynamics

The red dotted lines depict the relation between trunk angle and trunk moment of each individual over the entire two-minute trial. The fifteen subjects depicted on the left side are subjects without low back pain (CTRL), the fifteen subjects on the right are subjects with chronic low back pain (CLBP). The slope of the solid black line is identical to the stiffness obtained using the optimization procedure of the forward dynamic model used in this study. The dotted black line is the regression line between trunk angle and trunk moment.

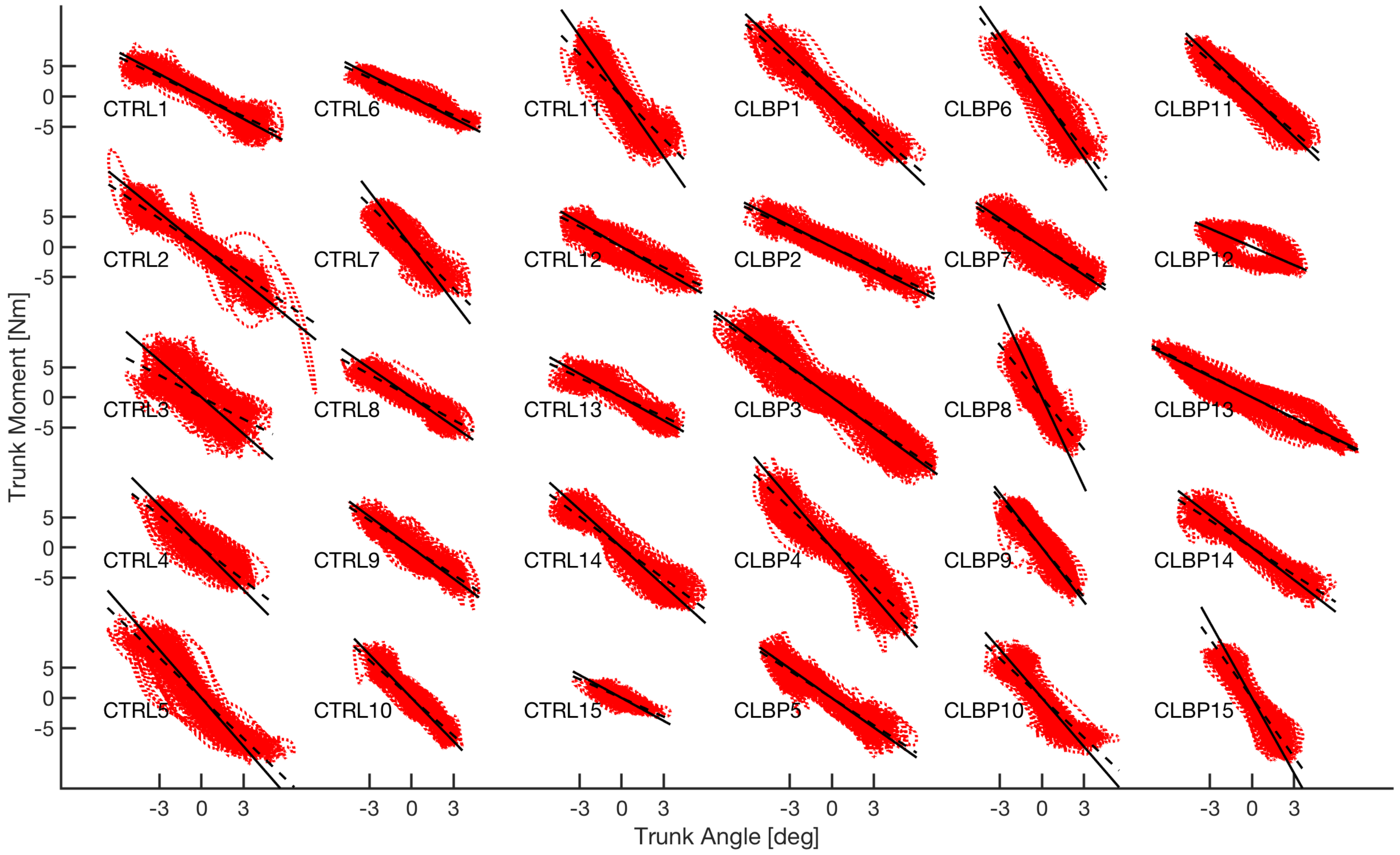

CTRL1
CTRL6
CTRL11
CLBP1
CLBP6
CLBP11
CTRL2
CTRL7
CTRL12
CLBP2
CLBP7
CLBP12
CTRL3
CTRL8
CTRL13
CLBP3
CLBP8
CLBP13
CTRL4
CTRL9
CTRL14
CLBP4
CLBP9
CLBP14
CTRL5
CTRL10
CTRL15
CLBP5
CLBP10
CLBP15
Trunk Moment [Nm]
Trunk Angle [deg]
